# 3D printing of bone and cartilage with polymer materials

**DOI:** 10.3389/fphar.2022.1044726

**Published:** 2022-12-06

**Authors:** Daoyang Fan, Yafei Liu, Yifan Wang, Qi Wang, Hao Guo, Yiming Cai, Ruipeng Song, Xing Wang, Weidong Wang

**Affiliations:** ^1^ Department of Orthopedic, The First Affiliated Hospital of Zhengzhou University, Zhengzhou University, Zhengzhou, China; ^2^ Department of Additive Manufacturing, The First Affiliated Hospital of Zhengzhou University, Zhengzhou, China; ^3^ Department of Pediatrics, The Third Affiliated Hospital of Zhengzhou University, Zhengzhou University, Zhengzhou, China; ^4^ Beijing National Laboratory for Molecular Sciences, Institute of Chemistry, Chinese Academy of Sciences, Beijing, China; ^5^ University of Chinese Academy of Sciences, Beijing, China

**Keywords:** bio printing, 3D printing, polymer materials, bone, cartilage

## Abstract

Damage and degeneration to bone and articular cartilage are the leading causes of musculoskeletal disability. Commonly used clinical and surgical methods include autologous/allogeneic bone and cartilage transplantation, vascularized bone transplantation, autologous chondrocyte implantation, mosaicplasty, and joint replacement. 3D bio printing technology to construct implants by layer-by-layer printing of biological materials, living cells, and other biologically active substances *in vitro*, which is expected to replace the repair mentioned above methods. Researchers use cells and biomedical materials as discrete materials. 3D bio printing has largely solved the problem of insufficient organ donors with the ability to prepare different organs and tissue structures. This paper mainly discusses the application of polymer materials, bio printing cell selection, and its application in bone and cartilage repair.

## 1 Introduction

Damage and degeneration to bone and articular cartilage are the leading causes of musculoskeletal disability (Zhang et al., 2022a; Zhu et al., 2022; Siegel et al., 2021). Articular cartilage is a kind of hyaline cartilage rich in type II collagen and proteoglycan, which plays an essential role in joint activities by carrying mechanical loads and lubricating joints. Unlike most tissues, articular cartilage has no blood vessels, nerves, or immune responses. Within its tissue structure, its ability to self-repair after degeneration or injury is minimal (Zhang et al., 2022a). Most of the large segment of bone and cartilage damage caused by trauma, disease, or tumor resection exceeds the self-healing ability of the bone and requires surgical repair and reconstruction. Commonly used clinical and surgical methods include autologous/allogeneic bone and cartilage transplantation, vascularized bone transplantation, autologous chondrocyte implantation, mosaicplasty, and joint replacement (Bei et al., 2021). The bone mentioned above and cartilage transplantation repair methods have problems such as limited tissue in the donor site, additional surgical damage, and disease transmission. Joint replacement is only suitable for advanced cartilage degeneration, and the prosthesis is expensive. 3D bio printing technology to construct implants by layer-by-layer printing of biological materials, living cells, and other biologically active substances *in vitro*, which is expected to replace the repair mentioned above methods. 3D printing technology plays a massive role in the biomedical field because of its unique advantages (Critchley et al., 2020). Researchers use cells and biomedical materials as discrete materials. 3D bio printing has largely solved the problem of insufficient organ donors with the ability to prepare different organs and tissue structures (Critchley et al., 2020; Ruiz-Cantu et al., 2020; Liu et al., 2021a; Potyondy et al., 2021). Especially in the application of bones and bone scaffolds, the advent of 3D printing technology provides a solution for treating patients with complex bone defects. The intersection of 3D printing technology and the field of biomedicine will indeed become a highlight of modern medicine.

Tissue engineering aims to develop natural tissue-mimicking three-dimensional porous composite scaffolds as frameworks for cell migration, adhesion, and growth to replace damaged tissue (Su et al., 2021). Cells seeded in traditional tissue engineering scaffolds can only be attached to the surface of the scaffold, and the distribution and migration of cells inside the scaffold cannot be precisely controlled, thus affecting its clinical effect (Yang et al., 2021a). In the past decade, 3D bio printing technology has developed rapidly in regenerative medicine. This technology can simultaneously combine living cells, extracellular matrix, and other biomaterials to construct 3D artificial implants or complex biological tissues through customized additive manufacturing. 3D bio printing technologies include inkjet bio printing/droplet bio printing, extrusion bio printing, and laser-assisted bio printing. In bone and cartilage tissue engineering, the main advantage of 3D bio printing is that it can print scaffolds with controlled distribution of cells, promoting cartilage tissue regeneration. In addition to the limitations of its technology in the application process, the choice of printing materials also plays a crucial role (De Angelis et al., 2021; Jang et al., 2020; Fan et al., 2020; Cao et al., 2021; Golebiowska and Nukavarapu, 2022). Materials currently used in 3D printing natural bones and bone scaffolds include metal materials, inorganic non-metallic materials, and polymer materials. Among them, polymer materials are widely used in the preparation of various organizational engineering alloys. Compared with metal and ceramics, polymer materials have excellent design flexibility because their composition and structure can be customized according to specific needs. Through molecular design, polymer materials can have the characteristics of hydrolyzed or enzymatic dismissal, which is more conducive to its metabolism in the body. At the same time, biologically active factors, drugs, or proteins can be carried out by side chain decoration so that the polymer biomaterial has better tissue regeneration capabilities. Commonly used polymers are divided into two types: natural polymer and synthetic polymer. Natural polymers include hyaluronic acid, gum, alginate, shell polycation, and collagen obtained from natural tissue polymers. Synthetic polymers include artificial synthetic polymers such as poly lactic-co-glycolic acid (PLGA), polymethylene acrylic (PMMA), PCL, and polyethylene glycol (PEG).

**FIGURE 1:**
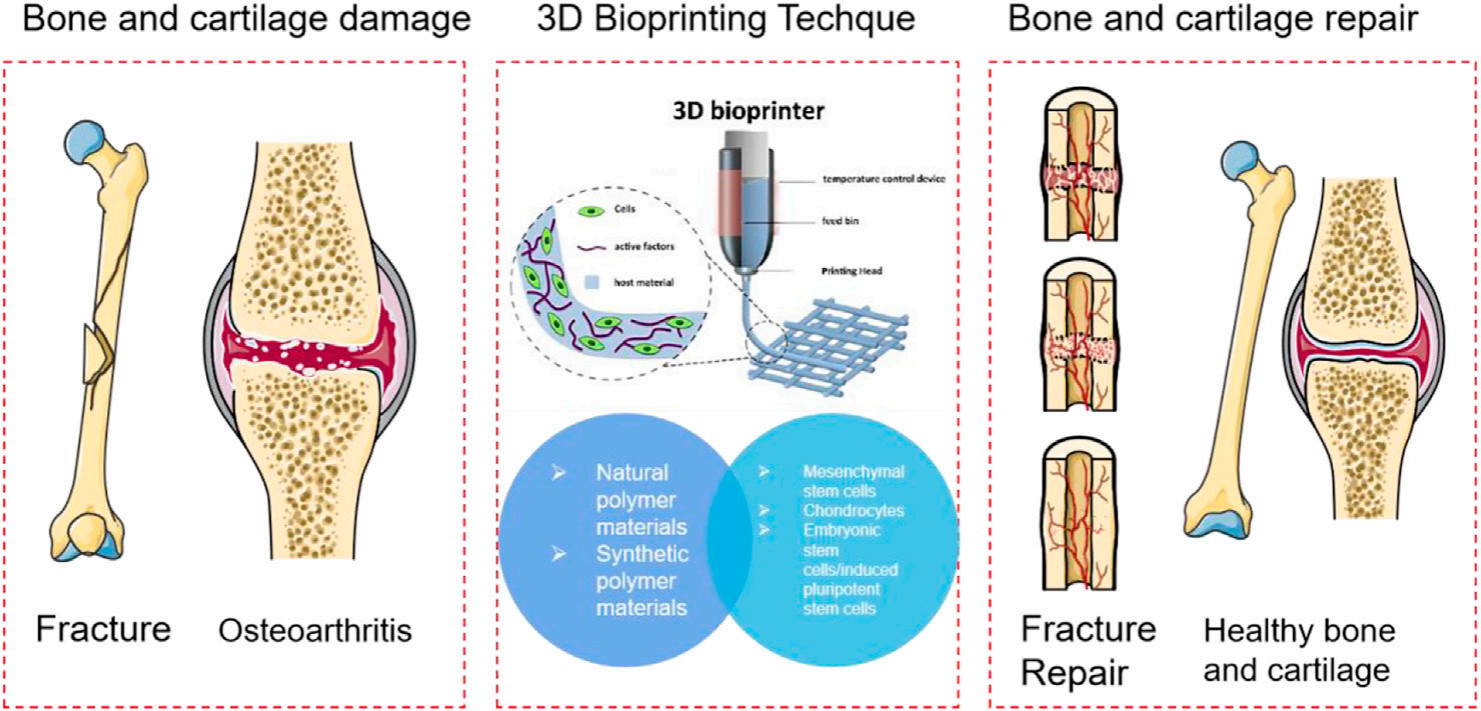
Schematic diagram of bone and cartilage using 3D bio printing based on polymer materials.

Polymer materials are usually fabricated with biologically squeezed 3D printing techniques. Natural polymer materials and partial synthetic polymer materials are easy to be prepared into hydrogel biological ink. Its hydrophilic capacity and biocompatibility are close to biological tissue. The mechanical properties can be adjusted, forming a stable three-dimensional structure. Most reported biological inks are primarily used in the gas phase environment *in vitro*. Even if liquid-phase suspended 3D printing is currently studied, it could only be printed in a specific liquid-phase solvent. The ingredients in the actual application environment of the biomaterial are complicated. Different printed biomaterial components and cells will create different properties and functions. This study focuses on the selection of polymer materials, biological printing cells, and their application in bone and cartilage repair. In detail, this review reveals each component’s characteristics used to carry out the basis and progress of 3D printing applications in the biological bone and cartilage problems. (Figure 1; Table 1).

**TABLE 1:** Polymer materials and cells in the application of 3D printing of bone and cartilage with their general properties.

^Material Types^	^Polymers^	^Bioactive content^	^Cells^	^Printing Techniques^	^General properties^	^References^
^Natural polymer materials^	^Collagen^	^*Prionace glauca* (PG), β-TCP, GelMA^	^Fibroblast Cell Line, Marrow-Derived Mesenchymal Stem Cells (mscs), Pdl Cell^	^Extrusion-Based Bioprinting^	^Non-cytotoxicity, low antigenicity response, crosslinking capacity, enzymatic biodegradability, complex structure^	^Heinrich et al. (2019;^ ^Diogo et al. (2020);^ ^Lin et al. (2021);^ ^Enrico et al. (2022)^
	^Sodium alginate^	^Carbohydrazide, silk fibroin, carbon nanotubes^	^Bmsc, Skin And Bone Cell Lines^	^Extrusion-Based Bioprinting^	^Complex-shaped, good biocompatibility, no immune rejection, good mechanical properties^	^Heo et al. (2020);^ ^Ni et al. (2020);^ ^Kim et al. (2021a);^ ^Gao et al. (2021);^ ^Khoshnood et al. (2021)^
	^Chitosan^	^cellulose nanofibrils (TCNFs), casein, dextran^	^Primary Osteoblast Cells, Encapsulated mscs^	^Extrusion-Based Bioprinting^	^Excellent performance, non-toxic, biocompatibility, and biodegradability, poor mechanical properties^	^Lee et al.(2013);^ ^Akkineni et al. (2016)^ ^Yang et al. (2018);^ ^Hafezi et al. (2020);^ ^Hu et al. (2020);^ ^Shen et al. (2020);^ ^Yang et al. (2021b);^ ^Castillo-Henríquez et al. (2021);^ ^Yang et al. (2021c);^ ^Poongodi et al. (2021);^ ^Biranje et al. (2022);^ ^Nakielski et al. (2022)^
	^Silk Fibroin^	^Gelatin, collagen, TGF-β3^	^Neural Stem Cells (Nscs)^	^Extrusion-Based Bioprinting^	^Good biocompatibility, non-toxicity, biodegradability, slow degradation rate insufficient mechanical properties^	^Jiang et al. (2020a);^ ^Sakai et al. (2020);^ ^Zhang et al. (2021b);^ ^Guo et al. (2021);^ ^Trucco et al. (2021);^ ^Rajput et al. (2022)^
	^Hyaluronic acid^	^Polysulfated glycosaminoglycan (PSGAG), transforming growth factor-β(TGF-β)^	^mscs, chondrocytes^	^Extrusion-Based Bioprinting^	^Excellent biocompatibility, biological activity and natural degradability^	^Huang et al. (2022a);^ ^Wang et al. (2022a);^ ^Wang et al. (2022b);^ ^Janarthanan et al. (2022);^ ^Kim and Lee. (2022);^ ^Martyniak et al. (2022)^
^Synthetic polymer materials^	^Polyetheretherketone^	^calcium hydroxyapatite (cHAp), amorphous magnesium phosphate^	^Primary Osteoblast Cells, Bmsc^	^Fused Filament Fabrication^	^Radiation permeability, no artifacts in magnetic resonance scanning, good biocompatibility, suitable elastic modulus^	^Gao et al. (2020;^ ^Oladapo et al. (2020);^ ^Prechtel et al. (2020);^ ^Yang et al. (2020);^ ^Puertas-Bartolomé et al. (2021);^ ^Sharma et al. (2021);^ ^Zheng et al. (2021);^ ^Zhu et al. (2021);^ ^Wang et al. (2022c)^
	^Polylactic acid^	^hydroxyapatite^	^Fibroblasts, Chondrocytes^	^Fused Filament Fabrication^	^Renewable resources, biodegradable, biocompatibility, gloss and transparency, mechanical properties, degradability, low melting point, and low viscosity^	^He et al. (2020b);^ ^Grottkau et al. (2020);^ ^Chang et al. (2021);^ ^Custodio et al. (2021);^ ^Wang et al. (2022d);^ ^Golebiowska and Nukavarapu, (2022);^ ^Rodríguez-Merchán, (2022);^ ^Waldburger et al. (2022)^
	^Polycaprolactone^	^Hydroxyapatite, vascular endothelial growth factor, (VEGF)^	^Human Umbilical Vein Endothelial Cells (Huvecs), Hbmscs^	^Fused Filament Fabrication^	^Biodegradable, processability, good mechanical properties, high crystallinity, low melting point, excellent rheological properties^	^Bandyopadhyay et al. (2020);^ ^Mousavi Nejad et al. (2021);^ ^Nulty et al. (2021);^ ^Li et al. (2022b);^ ^Koch et al. (2022);^ ^Xu et al. (2023)^
	^Polyamide^	^Hydroxyapatite, carbon nanotubes (CNTs), graphene nanoplatelets (GNPs)^	^mscs^	^sintering-based 3D printing, Multi Jet Fusion (MJF)^	^Biocompatibility,^	^Powell et al. (2020);^ ^Poltorak et al. (2021);^ ^Tey et al. (2021);^ ^Zhang et al. (2022b);^ ^Helal et al. (2022)^
	^Polylactic acid-glycolic acid copolymer^	^Mg^	^Embryonic stem cells (ESCs), Chondrocytes, Synovial MSCs (SMSCs)^	^low-temperature rapid prototyping (LT-RP)^	^Non-toxicity, good biological activity, biocompatibility, mechanical properties^	^Jiang et al. (2020b);^ ^Li et al. (2021b);^ ^Koons et al. (2021);^ ^Long et al. (2021)^
	^Conductive polymers^	^silver network, carbon nanofiber (CNF)^	^3T3 cells, mscs^	^FDM, MJF, Polyjet^	^Conductive, biocompatibility,^	^Serafin et al. (2021);^ ^Zhang et al. (2022c);^ ^Ding et al. (2022);^ ^Gong et al. (2022);^ ^Murab et al. (2022);^ ^Zamboni et al. (2022)^
	^Photosensitive resin^	^rubber contents, epoxy polymer, reactive diluent^	^mscs^	^UV-curing^	^Crosslinking capacity,^	^Fang et al. (2020);^ ^Shan et al. (2020);^ ^Bud et al. (2021);^ ^Liu et al. (2021d);^ ^Fleck et al. (2021);^ ^Hua et al. (2021);^ ^Mahmoudi et al. (2021);^ ^Dadras-Toussi et al. (2022b);^ ^Huang et al. (2022b)^

## 2 Polymer materials for 3D bio printing of bone and cartilage

### 2.1 Natural polymer materials

#### 2.1.1 Collagen

Collagen is the most important structural protein in human tissue and an essential component of the extracellular matrix, which is the main component of the cartilage matrix. Collagen molecules have been widely used in biomedical applications due to their weak antigenicity, degradability, excellent biocompatibility, and biomimetic functions (Wang et al., 2019; Burdis et al., 2021). The scaffold prepared with collagen as raw material benefits cell adhesion and supports and protects cells (Dai et al., 2021; Lee et al., 2021). However, collagen also has disadvantages, such as no melting point, low denaturation temperature, insoluble water, high viscosity, low mechanical stability, fast degradation speed, insufficient mechanical strength, *etc.* These deficiencies are mainly solved by compounding collagen with other materials.

**FIGURE 2:**
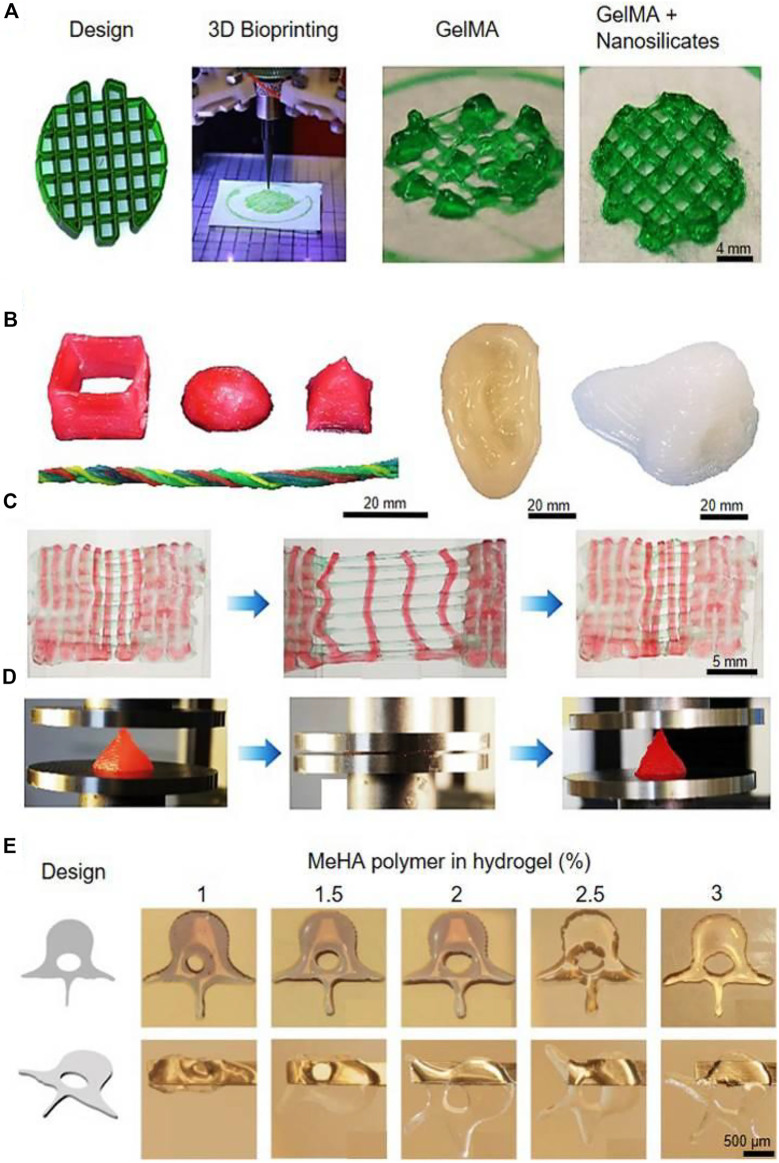
**(A)** Bioprinting of GelMA for rigid complex structures. **(B)** Various 3D constructs were bioprinted with a PEG hydrogel. **(C)** Bioprinted PEG undergoing repeated stretching and recovering tests. **(D)** Bioprinted PEG undergoing repeated compressions and recovering tests. **(E)** Human spine vertebrae bioprinted with concentrations of MeHA in the bioink. Reproduced with permission from ref (Heinrich et al., 2019).

Diogo et al. (2020) proposed *in situ* mineralization of blue shark [*Prionace glauca* (PG)] collagen to fabricate 3D printable cell-laden hydrogels. Mouse fibroblast cell line survival during and after printing was favored by the presence of PG collagen as exhibited by the biological performance of the hydrogels. Enrico et al. (2022) rearranged the collagen fibers with laser irradiation of the hydrogel to generate cavitation gas bubbles, thereby creating stable micro channels. It enables organs-on-a-chip and 3D tissue models featuring complex. Lin et al. (2021) developed a biomimetic microfibrous system capable of preparing collagen-based straight and waveform microfibers to guide PDL cell growth. 3D-printed collagen-based waveform microfibers preserved PDL cell viability and exhibited an enhanced tendency to promote healing and regeneration under shear stress. Liu et al. (2021a) designed a bone marrow-derived mesenchymal stem cell (BMSC)-laden 3D-bioprinted multilayer scaffold with methacrylate hyaluronic acid/polycaprolactone incorporating ketogenic and β-TCP for osteochondral defect repair within each region. BMSC-laden scaffolds facilitated chondrogenesis by promoting collagen II and suppressed interleukin 1β in osteochondral defects of the femoral trochlea. Congruently, BMSC-laden scaffolds significantly improved the joint function of the injured leg with respect to the ground support force, paw grip force, and walk gait parameters (Figure 2) (Heinrich et al., 2019).

#### 2.1.2 Sodium alginate

Alginate is a natural polysaccharide compound extracted from seaweed (Piras and Smith, 2020). It has excellent adhesion, good biocompatibility, and biodegradability unmatched by other materials (Olmos-Juste et al., 2021; Rastin et al., 2021). Salt has been widely used and developed in the field of biomedicine (Zhang et al., 2021a). Animal experiments have shown that high-purity sodium alginate has good biocompatibility, and no immune rejection occurs when implanted in animals (Chen et al., 2020; Erkoc et al., 2020; Gao et al., 2021). Heo et al. (2020) synthesized and deposited carbohydrazide-modified gelatin (Gel-CDH) into a new multifunctional support bath consisting of gelatin microparticles suspended in an oxidized alginate (OAlg) solution. They offered a novel strategy for bioprinting of natural polymer-based hydrogels into 3D complex-shaped biomimetic constructs. With other procedures, 3D-printed alginate scaffolds were coated by branch polyethylenimine to obtain branch with a large number of active N-H groups (Ni et al., 2020; Khoshnood et al., 2021). To induce rapid gelation, alginate derivatives were synthesized and mixed with silk fibroin. It revealed enhanced cell compatibility (Kim et al., 2021a). Besides with silk fibroin, carbon nanotubes were manufactured into cylindrical scaffolds through the collaboration to fabricate the hybrid bioink with alginate. Verified by mouse models, the proper doping of carbon nanotubes could effectively increase the mechanical properties of composite scaffolds.

#### 2.1.3 Chitosan

Chitosan is a kind of biological material with abundant resources and excellent performance, non-toxic, biocompatibility, and biodegradability (Demirtaş et al., 2017; Yang et al., 2022). It is an ideal extracellular matrix material that can promote various tissue—cell adhesion and proliferation (Xu et al., 2022). Chitosan has biological activity, which can promote the growth of vascular endothelium, and the proliferation of keratinocytes and osteoblasts, and also has the properties of anti-inflammatory, antibacterial, and immune function regulation. Chitosan has been used as a growth factor carrier and scaffold material in the skin, nerve, bone and cartilage, and liver tissue engineering and can also be used as wound dressings, drug release agents, and defect fillers (Liu et al., 2021b; Liu et al., 2021c; Neufurth et al., 2021). However, scaffolds prepared from pure chitosan also have shortcomings, such as poor mechanical properties and lack of material surface specificity (Demirtaş et al., 2017; Liu et al., 2021b). Therefore, when chitosan is used in bone tissue engineering, it is usually compounded with other materials to achieve the required performance requirements (Yang et al., 2018; Yang et al., 2021b; Yang et al., 2021c). Biranje et al. (2022) designed and fabricated a 3D composite scaffold with cellulose nanofibrils (TCNFs), chitosan, and casein. This scaffold can accelerate blood clotting and wound healing, suggesting its potential application in reducing blood loss during traumatic hemorrhage. Alginate, chitosan, gellan gum, gelatin, and collagen hydrogels were utilized successfully as core materials-hydrogels which are too soft for 3D plotting of open-porous structures without an additional mechanical support (Akkineni et al., 2016). Although chitosan is abundant in nature, has excellent properties, and is environmentally friendly, its mechanical properties are poor, which limits its application of chitosan. Chitosan has relatively active free radical groups and is relatively active (Lee et al., 2013; Hafezi et al., 2020; Hu et al., 2020; Castillo-Henríquez et al., 2021). It can also be modified by chemical reagents to prepare corresponding composite materials, further expanding the chitosan application field (Shen et al., 2020; Poongodi et al., 2021; Nakielski et al., 2022).

**FIGURE 3:**
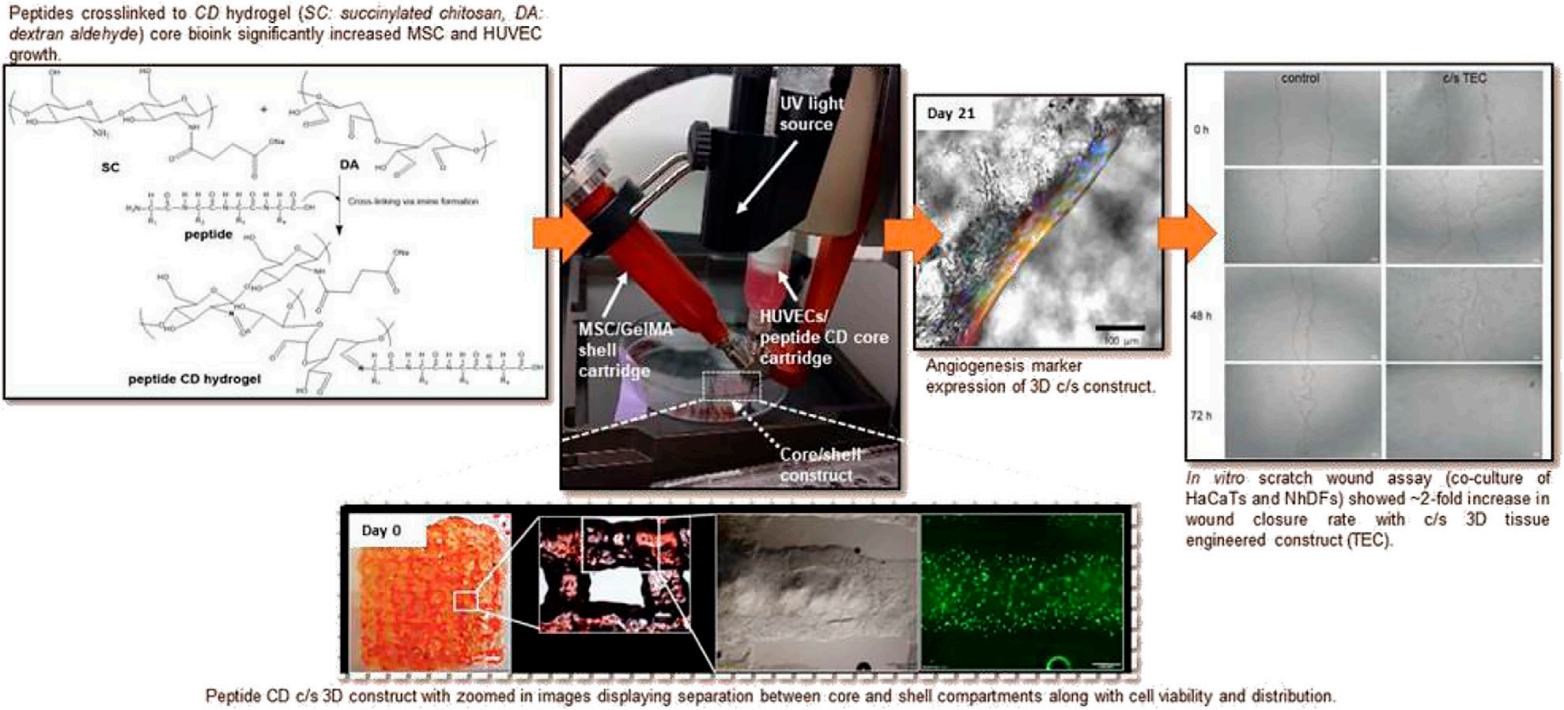
Peptide chitosan/dextran core/shell vascularized 3D constructs for wound healing. Reproduced with permission from ref (Yang et al., 2021c).

Combining the contents of natural and synthetic polymers, it is not difficult to find that polymer materials for biological tissue engineering should meet the following requirements: 1) Good biocompatibility; 2) Controllable degradability; 3) Mechanical property; 4) Self-growth performance and 5) Good sterilization (Figure 3) (Yang et al., 2021c).

#### 2.1.4 Silk fibroin

Silk fibroin is natural macromolecular fibrin with excellent physical and chemical properties (Kim et al., 2021a; Kim et al., 2021b). Silk fibroin has the following advantages: good biocompatibility, non-toxicity, biodegradability, no toxic and side effects of degradation products, slow degradation rate *in vivo*, and its degradation rate can be adjusted by changing its structural form (Ni et al., 2020; Deng et al., 2021; Guo et al., 2021). Because of its unique and excellent properties, silk fibroin has shown great application prospects in biomedicine (Ni et al., 2020; Sakai et al., 2020; Kim et al., 2021b; Rajput et al., 2022). However, pure silk fibroin has insufficient mechanical properties. The modification of silk fibroin by compounding it with other materials can improve silk fibroin. Silk fibroin-gelatin (G)-based hydrogel was fabricated as an analytical model to predict the extruded filament width in order to maximize the printed structure’s fidelity to the design. In this study, with gelatin mixed with silk fibroin, hydrogel revealed ability to achieve more controlled and standardized products than classical trial-and-error approaches in the biofabrication of engineered constructs (Trucco et al., 2021). In another study, a type of three-dimensional collagen/silk fibroin scaffold (3D-CF) was fabricated with cavities that simulate the anatomy of normal spinal cord. With transplantation of neural stem cells (NSCs), 3D-CF combined with NSCs can promote the repair of injured spinal cord (Jiang et al., 2020a). In another interesting study, bioinks with different concentrations of silk fibroin and decellularized extracellular matrix (SF-dECM) was prepared and mixed with bone marrow mesenchymal stem cells (BMSCs) for 3D bioprinting. With releasing TGF-β3, the SF-dECM had the ability to promote chondrogenic differentiation of BMSCs and provided a good cartilage repair environment, suggesting it is an ideal scaffold for cartilage tissue engineering (Zhang et al., 2021b).

#### 2.1.5 Hyaluronic acid

Hyaluronic acid is a linear anion glycosamine in vertebrates’ tissue and body fluid. Hyaluronic acid was first separated from the ox eye vitreous in 1934, and its structure was proposed in 1970 (Desai et al., 2022). Hyaluronic acid is abundant in the extracellular matrix of the human embryo and connective tissues. It also exists in a considerable amount to play a role in nutrition, lubrication, and shock absorption on joints (Flégeau et al., 2022). Hyaluronic acid has unique physical-chemical properties and excellent biocompatibility, biological activity, and natural degradability. Its three-dimensional honeycomb structure pore rate is high, and ample internal space and surface area are conducive to adhesion, proliferation, and seed cell differentiation (Desai et al., 2022). Hyaluronic acid can be combined with CD44. By inhibiting the expression of interleukin 1β, causing matrix metal prop (MATRIX Metalloproteire-1, MMP-1), MMP-2, MMP-3, MMP-9, and MMP-13 Synthesis decreases, reduce the activity of alien enzymes in arthritis, and promote the proliferation of cartilage cells while lowering the apoptosis of cartilage cells and protecting cartilage (Huang et al., 2022a). Studies have shown that the bracket with hyaluronic acid as the material has apparent bone induction effects during the cartilage repair process, which can significantly promote the repair of defects. It has broad application prospects in the field of tissue engineering cartilage repair (Janarthanan et al., 2022). Because natural hyaluronic acid is still inadequate in biocompatibility, biodegradation, mechanical strength, and host tissue integration, it cannot meet the requirements of excellent cartilage repair in organizational engineering. Kim and Lee (2022) reported a water-based polyurethane cluster bracket with hyaluronic acid as a carrier. This bracket is a bracket that is very close to joint cartilage. At the same time, 3D printing technology can be designed to match the three-dimensional structure of the bracket to match the matching. The shape of the cartilage defect provides the most effective way to repair and reconstruct the cartilage tissue. Martyniak et al. (2022), proposed the bionic cartoons of the bionic cartilage cells of the emulsion and branches of hyaluronic acid. Adding ultra-smooth magnetic iron oxide nanoparticles to the deeper shell shows good cell guidance capabilities and induced to induce it. The two physical stimuli of the static magnetic field and magnetic shear stress accelerated the regeneration of cartilage cells.

Active functional groups such as carboxyl, hydroxyl, and acetylamino in hyaluronic acid molecules can prepare new brackets by forming hydrogen bonds and other polymers by forming hydrogen bonds (Wang et al., 2022a). Point anion polysaccharide, so hyaluronic acid can be coupled with static electricity with cation polymers, which is another standard method for achieving hyaluronic acid composite modification. In recent years, an electric spinning nanoscope has been widely studied as a bracket material that promotes cell biological activity because it simulates collagen nano-fiber networks in an extracellular matrix (Kim and Lee, 2022). Wang et al. (2022a) synthesizes 3D-printed islet organoid by combining a pancreatic extracellular matrix (pECM) and hyaluronic acid methacrylate (HAMA). After research, the prepared brackets have no cytotoxicity, which can promote the adhesion, diffusion, and proliferation of seed cells. The above polymers show a significant synergy in regulating cartilage formation. A variety of polymer synthesis brackets can have more selectivity in the needs of their spatial structure design and physical and chemical properties. Wang et al. (2022b) develops a dual-factor-oriented porous structure of bionic cartilage. Made of sugar and sodium hyaluronate, prepared by collagen, chitosan, and sequin protein, the transition layer of the microtubule array structure is ready. Polyinine-sodium nanometer of heparin-sodium nanometer containing transformation growth factor β1, scanning electron microscopy shows that the double-layer composite bracket has a dual design similar to natural cartilage. At the same time, proliferation and differentiation, neonatal cartilage tissue, and surrounding tissue have achieved good integration, and the shape is the same as normal cartilage. Potyondy et al. 2021 synthesizes a three-phase hydrogel of collagen, condensate, and hyaluronic acid and then uses rabbits’ autologous cartilage cells for cartilage defect repair. Cell activity analysis and *in vitro* biochemical assessment show that cartilage cells in hyaluronic acid three-phase water express cell proliferation, and the expression of glycosamine secretion and cartilage differences in gel brackets are significantly higher than in ordinary gel.

### 2.2 Synthetic polymer materials

#### 2.2.1 Polyetheretherketone

Polyetheretherketone has the advantages of radiation permeability and no artifacts in magnetic resonance scanning, which can better evaluate postoperative recovery (Fairag et al., 2021; Han et al., 2022). It has been used in artificial joint jaws, skulls, and spines: lumbar spine, oral defect repair, and other fields (He et al., 2020a; Li et al., 2021a; Li et al., 2022a). Also, compared with traditional metal materials (stainless steel, titanium alloy) implanted into human body, polyetheretherketone has good biocompatibility, and its elastic modulus is comparable to human cortical bone (He et al., 2020a; Li et al., 2021a). It can effectively reduce the stress shielding effect after implantation into the human body. Polyetheretherketone has become the most promising artificial bone matrix composite material due to its excellent properties (Sikder et al., 2020). Medical polyetheretherketone is the best long-term bone graft material certified by the US Food and Drug Administration. Polyetheretherketone also has some disadvantages, such as no biological activity, low surface osteogenic efficiency (Prechtel et al., 2020).

**FIGURE 4:**
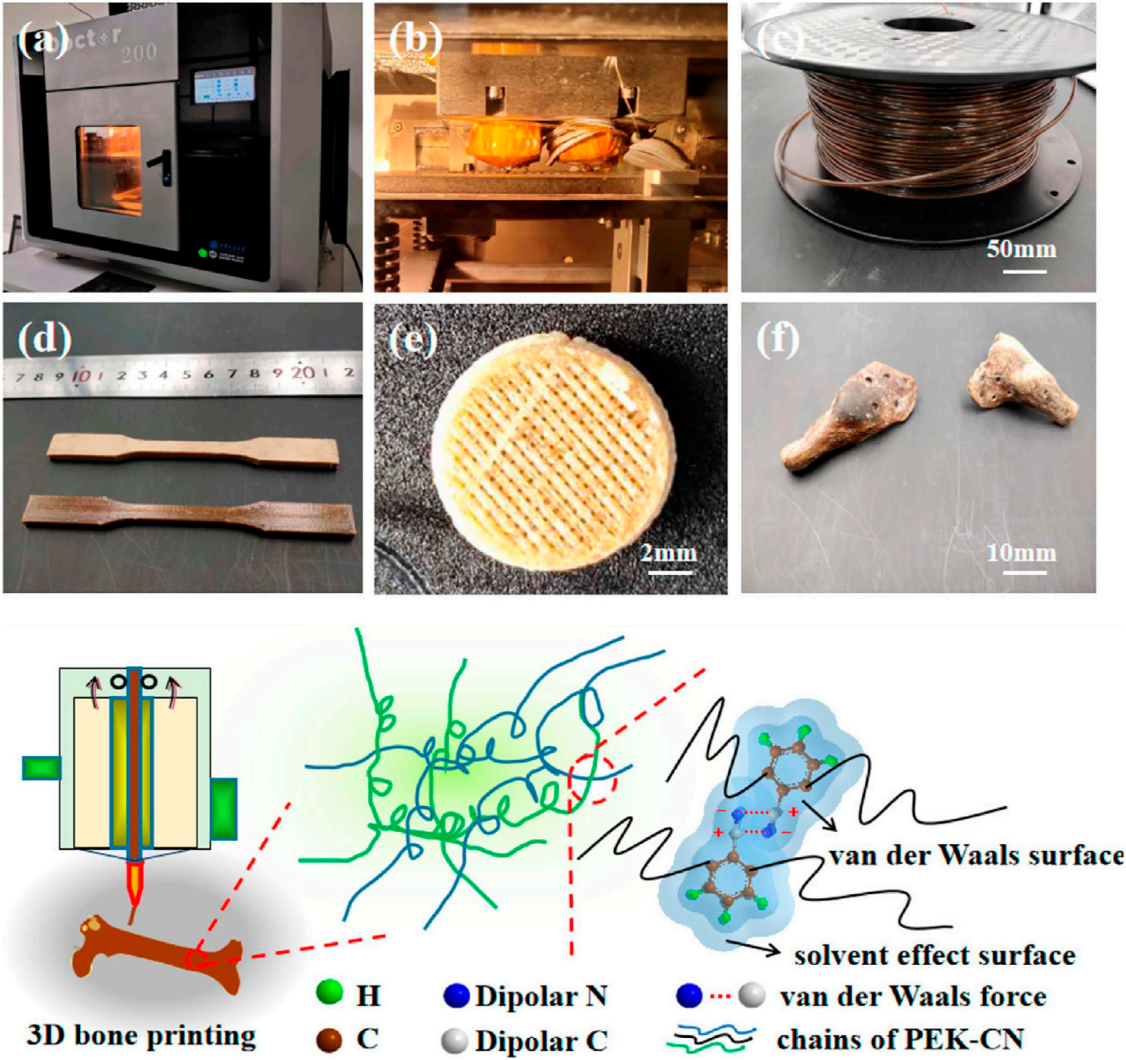
Amorphous poly (aryl ether nitrile ketone) and its composites with nano hydroxyapatite for printing artificial bone. Reproduced with permission from ref (Gao et al., 2020).

The additional biomaterials into PEEK such as calcium hydroxyapatite (cHAp) are effective ways to improve bone-implant interfaces and osseointegration. The PEEK/cHAp induced the formation of apatite after immersion in the simulated body fluid of DMEM for different days to check its biological bioactivity for an implant (Oladapo et al., 2020). In another study, novel amorphous magnesium phosphate (Puertas-Bartolomé et al., 2021) particles were mixed into PEEK to develop bioactive and osseointegrable dental and orthopedic implants. AMP-PEEK composites are good candidates for 3D printing by exhibiting high zero-shear and low infinite-shear viscosities (Sikder et al., 2020). Another promising composite is PEEK-HA. Here PEEK scaffolds with a series of hydroxyapatite (HA) contents in gradient were manufactured *via* fused filament fabrication (FFF) 3D printing techniques. Novel scaffolds exhibited higher Young’s modulus and lower compressive strength along Z printing direction. The mapping relationship among geometric parameters, HA content, printing direction, and mechanical properties was established, which gave more accurate predictions and controllability of the modulus and strength of scaffolds. The PEEK/HA scaffolds with the micro-structured surface can promote cell attachment and mineralization *in vitro* (Zheng et al., 2021). The composite of polyetheretherketone and inorganic non-metallic materials can improve the physical and chemical properties of artificial bones and artificial bone scaffolds and facilitate the spreading, adhesion, and growth of bone cells (Oladapo et al., 2020; Sikder et al., 2020; Yang et al., 2020; Zheng et al., 2021). Therefore, the composite of polyetheretherketone and inorganic materials in future development will become a new direction (Figure 4) (Gao et al., 2020; Sharma et al., 2021; Zhu et al., 2021; Wang et al., 2022c).

#### 2.2.2 Polylactic acid

Polylactic acid (PLA) is an aliphatic polyester, which can be extracted from renewable plant resources (such as corn and potato). Starch in renewable resources is used as raw material to obtain lactic acid through biological fermentation and following monomer polymerization (Aihemaiti et al., 2022; Buj-Corral et al., 2022). Polylactic acid can be converted into carbon dioxide and water in both nature and living organisms and is a truly environmentally friendly new biodegradable material (Chang et al., 2021; Wang et al., 2022d). The main reason why polylactic acid can be used as a 3D printing material is that it has good biocompatibility, gloss and transparency, mechanical properties, degradability, low melting point, and low viscosity (Chang et al., 2021; Wang et al., 2022d). While it has defects such as greater brittleness and poor impact resistance (Custodio et al., 2021). A series of multi-zonal and gradient structures were fabricated with bi-phasic and tri-phasic configurations. Polylactic acid (PLA) was used for the fabrication of zonal/gradient scaffolds to provide mechanical strength. It revealed structural hierarchy and mechanical integrity for bone-cartilage interface engineering (Golebiowska and Nukavarapu, 2022).

Moreover, a highly porous scaffold with anatomical-shape characteristics was fabricated with polylactic acid polymer (PLA) and PLA-hydroxyapatite (HA). The HA-incorporated scaffolds demonstrated significantly higher compressive strength, modulus, and osteoinductivity as evidenced by higher levels of alkaline phosphatase activity and calcium deposition (Grottkau et al., 2020). PLA polymer struts on a nanofiber web to fabricate a nanoporous filter with a hierarchical structure and transparent look. The transparent look overcomes the threatening appearance of the masks which can be a feasible way of reducing the social trauma caused by the current CoV disease-19 pandemic (He et al., 2020b). Three gellan gum-graft-poly (d,l-lactide-co-glycolide) copolymers (GGm-PLGA) which differed in the graft substitution degree were synthesized and characterized. It revealed that fibroblasts and chondrocytes remained viable after printing and over a culture period of 7 days into scaffolds (Rodríguez-Merchán, 2022). From studies to clinical applications, twenty patients were evaluated for the general applicability and possible benefit of PLA in the immobilization of hand surgery patients. It suggested that 3D-printed splinting is feasible and satisficed in clinical applications (Waldburger et al., 2022).

#### 2.2.3 Polycaprolactone

Polycaprolactone (PCL) is a biodegradable polyester with good biocompatibility and non-toxicity (Chiesa-Estomba et al., 2021). As a biodegradable medical material, it is widely used in the medical field. Processability and good mechanical properties, high crystallinity and low melting point, excellent rheological properties, and viscoelasticity endow it with good melt printing ability (Cui et al., 2021; Hamid et al., 2021). Polycaprolactone also can store and restore deformation, It can adapt to the rapid development of 3D printing technology, is suitable for making tissue engineering scaffolds, and becomes a common material for biological 3D printing (Koch et al., 2022; Xu et al., 2023). Microstructural scaffolds designed with polycaprolactone as raw materials can provide structural support and transport channels to induce tissue regeneration (Li et al., 2022b). They can also serve as sites for cell adhesion, proliferation, and differentiation, providing a suitable physical environment for newly formed tissues.

**FIGURE 5:**
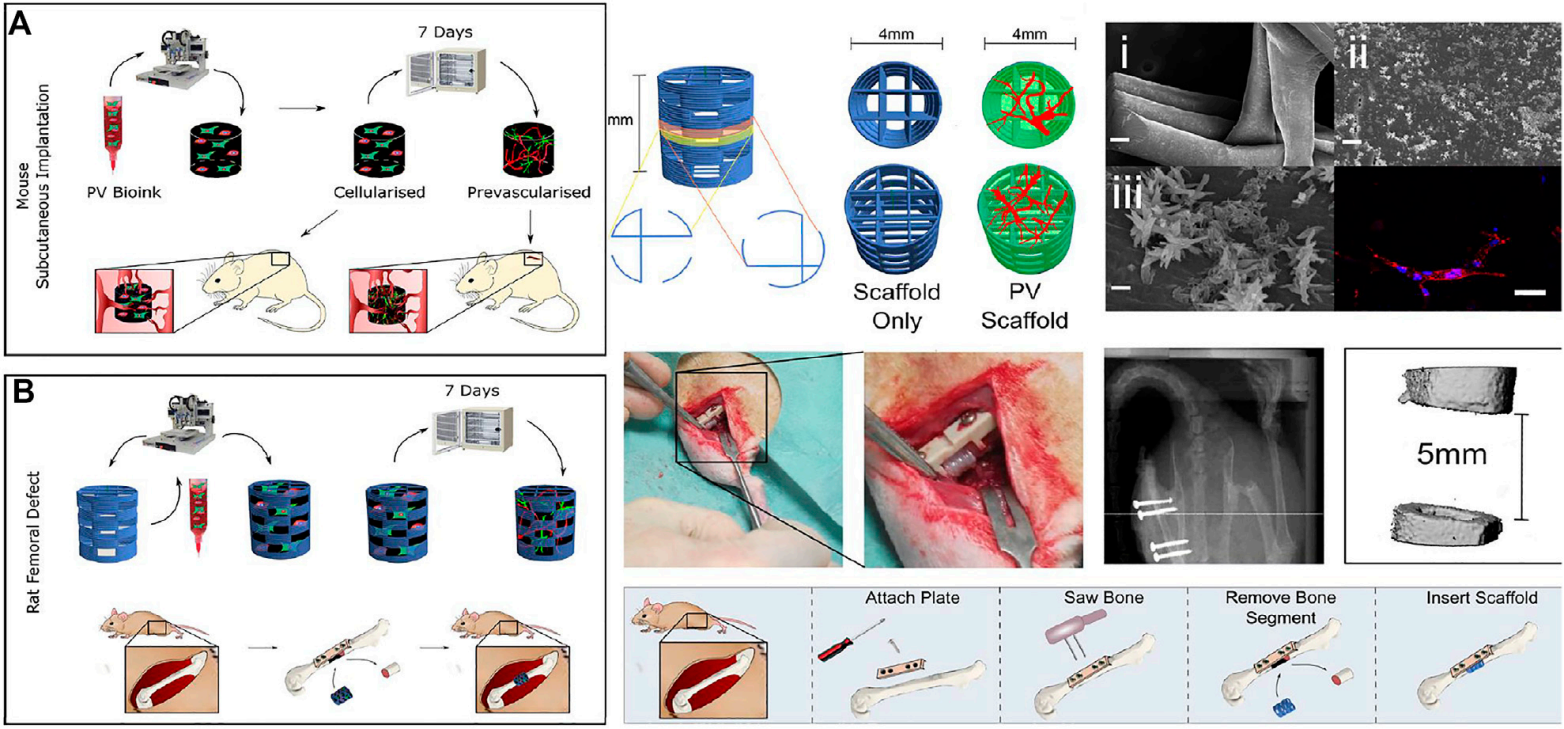
The fibrin-based hydrogel was applied with the HUVEC microvessel network in bio printing for bone regeneration with application in subcutaneous tissue **(A)** and femoral defect **(B)** in mouse. Bioink was combined with HUVECs and supporting human bone marrow stem/stromal cells (hBMSCs). Reproduced with permission from ref (Nulty et al., 2021).

However, polycaprolactone scaffolds also have shortcomings like poor adhesion (Bandyopadhyay et al., 2020). Researchers improved the performance of scaffolds by blending polycaprolactone with other materials. For example, PCL/HA was fabricated by 3D printing technology (Bandyopadhyay et al., 2020). The surface treatment of the PCL scaffold with HA considerably increased the hydrophilicity of the scaffolds which led to an enhancement in cell adhesion (Mousavi Nejad et al., 2021). A bioprinting strategy to engineer vascularized tissues was developed with PCL. The capacity to enhance the vascularisation and regeneration of large bone defects *in vivo* was enhanced with co-bioprinted containing both HUVECs and hBMSCs (Waldburger et al., 2022). In another study, 3D bioprinting was applied with HUVECs and supporting hBMSCs in the fabrication for potental reconstruction (Figure 5) (Nulty et al., 2021).

#### 2.2.4 Polyamide

Polyamide, commonly known as nylon, is a common medical polymer with high polarity and exhibits excellent properties in terms of biocompatibility (Dann et al., 2021). The polyamide 66/nano-hydroxyapatite composite material combines the excellent properties of the two materials, has good mechanical properties, and has the biological activity of hydroxyapatite, which has great potential in the treatment of bone defects by 3D printing (Helal et al., 2022). Poltorak et al. (2021) designed and 3D printed a polyamide-based electrochemical cell with polyamide that was used as the liquid-liquid interface support during electroanalytical measurements. Switchable, photochromic tungsten oxide nanoparticles, which are colorless even at high concentrations was designed as fusing agents for polyamide powders in sintering-based 3D printing (Powell et al., 2020). Polyamide 11 (PA11) and thermoplastic polyurethane (TPU) powder were conducted in the 3D printing process of Multi Jet Fusion (MJF) (Tey et al., 2021). Furthermore, a polyamide 12-based thermoplastic composite was modified with carbon nanotubes (CNTs), CNTs grafted onto chopped carbon fibers (CFs), and graphene nanoplatelets (GNPs) with CNTs to improve its thermal conductivity for application as a heat sink in electronic components (Zhang et al., 2022b).

#### 2.2.5 Polylactic acid-glycolic acid copolymer

The polylactic acid-glycolic acid copolymer is widely used in medicine, chemistry, industry, and other fields because of its non-toxicity and good biological activity, biocompatibility, and mechanical properties (Barati et al., 2020; Carlier et al., 2021). The polylactic acid-glycolic acid copolymer can be degraded by breaking the ester bond, and its degradation products are the same as those of human metabolism (Jiang et al., 2020b). This method has been widely used in the biomedical field by adjusting the monomer ratio to change the degradation time of PLA-glycolic acid copolymer. The Food and Drug Administration in the United States has certified the polylactic acid-glycolic acid copolymer. It is officially included in the US Pharmacopoeia as a pharmaceutical excipient (Barati et al., 2020; Koons et al., 2021), but its degradation product will generate acid that may cause the potential inflammation (Li et al., 2021b). In tumor treatment, innovative PLGA/Mg porous scaffolds were fabricated for postsurgical management of osteosarcoma. PLGA/Mg composite scaffolds were fabricated with low-temperature rapid prototyping (LT-RP) 3D-printing technology. It revealed excellent biodegradability and biocompatibility, exhibiting great promise for clinical translation (Long et al., 2021).

#### 2.2.6 Conductive polymers

The conductive materials usually printed in 3D are made based on non-metallic 3D printing technology on the thin film base (Abbasi-Ravasjani et al., 2022). The appropriate 3D printing technology, dispersed liquid or conductive elastic composite material of the conductive filler, could be chosen for the flexible base to obtain the circuit pattern and the required conductive material and device (Abbasi-Ravasjani et al., 2022). The 3D printing technology involved in the manufacturing of conductive materials mainly includes melting deposition (FDM), electric field-driven spray deposition (E-JET), polymer injection molding (Polyjet), direct ink writing (DIW), stereo light carvings (SLA) (Abbasi-Ravasjani et al., 2022; Andjela et al., 2022; Ding et al., 2022). According to the characteristics of the material, the appropriate 3D printing technology can be selected to manufacture the conductive material. SLA and Polyjet are suitable for optical solid-cured resin materials. FDM is ideal for materials that easily squeeze out the small nozzle after heating and melting (Andjela et al., 2022). Polyjet, E-JET, and DIW Requirement materials have the changing characteristics of shear and dilute. Low viscosity should be shown at a high shear rate, like liquid, to allow the ink to squeeze through the detailed printing nozzle. And printing ink also needs to have high viscosity, showing a paste at a low shear rate to keep the shape after 3D printing without collapse (Gong et al., 2022).

Combined with the E-JET printing technology and hybrid hot pressure technology, Zamboni, F. proposed an embedded silver network manufacturing technolog y with no mold, template-free, and electroplating (Zamboni et al., 2022). A flexible transparent electrode with excellent photoelectric performance, mechanical stability, and environmental adaptability are prepared on the adjustable transparency base. The transparent electrode with transparent light transparency is excellent (Murab et al., 2022). Although these standard 3D printing processes can achieve higher-precision pattern electrodes and microstructure printing, this type of conductive material based on the point-to-line processing method is not only deficient in efficiency. The processing accuracy is related to the diameter of the nozzle of the extrusion material, so the higher the printing accuracy, the slower the printing speed. In contrast, digital light treatment (DLP) printing shows the advantages of high printing accuracy, fast relative velocity, and superior surface quality, which provides high-performance conductive material and devices for manufacturing negative perpopy pine ratio, complicated geometric shapes, and micro-surface structures (Murab et al., 2022).

**FIGURE 6:**
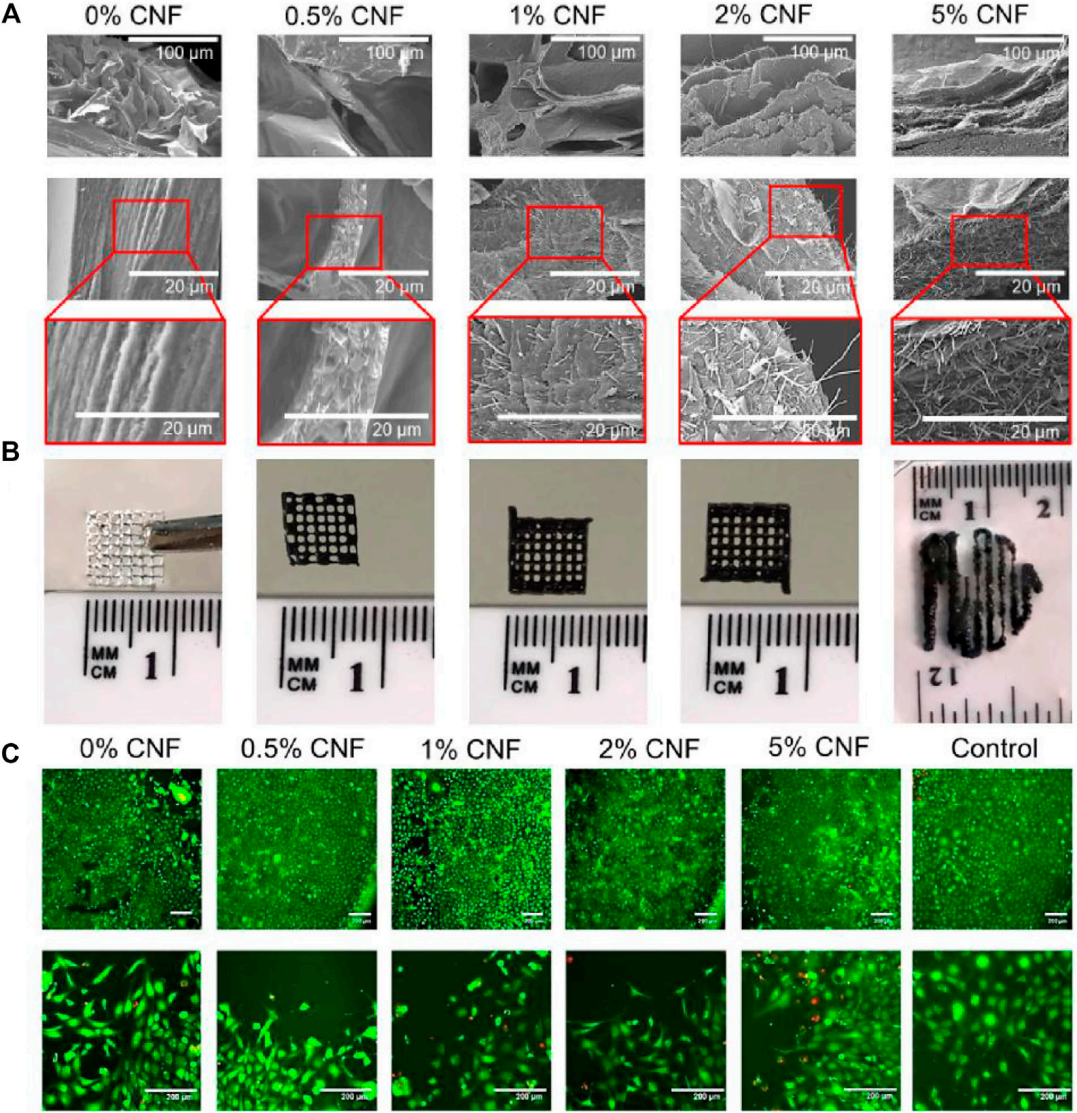
**(A)** SEM images of lyophilised hydrogels with different CNFs content. **(B)** Images of 3D printed lattices using alginate/gelatin/CNF hydrogels. **(C)** LIVE-DEAD assay. For all tests NIH/3T3 cells were cultured in the presence of the alginate/gelatin/CNF hydrogels over a period of 96 h. Reproduced with permission from ref (Serafin et al., 2021).

The hybrid printed biomass proposed by Professor Maurice N. Collins consists of algosate and gelatin hydrogel system containing carbon nanofiber (CNF) to create an electrons and printed 3D brackets (Serafin et al., 2021). It is important that the preparation method allows the formation of hydrogels with uniform dispersing CNF. Based on mechanical, chemical, and cell reactions, these hybrid composite material hydrogels were evaluated. The doping method can add electrical fillers to the optical elastic matrix, and the prepared, flexible body has a conductive function through physical or chemical processes (Figure 6) (Serafin et al., 2021). Mixed carboxy-based multi-wall carbon nanotubes (C-MWCNTS) to N-acrylceroprid (ACMO) resin can obtain good conductive nano-composite materials for DLP printing and strain sensors, which can be detected in real time and accurate detection human activity (Zhang et al., 2022c).

#### 2.2.7 Photosensitive resin

Photosensitive resin known as a photocurable solid material mainly consists of a photo-initiator, oligomer, and reactive diluent (Abdallah et al., 2020). The UV-Curing 3D Printing is the process of using the rapid cross-linking of liquid light-sensitive resin under ultraviolet light (UV) radiation to solid substances to solid substances (Dadras-Toussi et al., 2022a). So as to add optical curing light-sensitive resin layer by layer, until it forms a complete formation the process of three -dimensional device (Dadras-Toussi et al., 2022a). Because the light -sensitive resin has unique liquid liquidity and instantaneous light solidification characteristics and the amount of light sensitivity resin can accurately control the amount and space of the light sensitivity resin (Foster et al., 2022). The optical solidification 3D printing can create a prototype with any complex geometric shape that is difficult to prepare with traditional processing methods. The optical solidification 3D printing technology is the longest history, but the development speed is the fastest. It is also the most widely used type of 3D printing technology. It mainly includes SLA, DLP, LCD, CLIP and other methods (Foster et al., 2022). Its essence is a colloidal substance composed of macromolecules (Liu et al., 2021d). The molecules are scattered and cross-linked together like a fence. When the photosensitive resin is irradiated by ultraviolet light, the photo-initiator absorbs energy, forms excited molecules, and decomposes active groups (Bud et al., 2021). Moreover, it is manifested as the transformation of colloidal resin into a solid object. The light source in the 3D printer continuously cross-links the photosensitive resin by scanning layer by layer, thereby accumulating a three-dimensional solid product (Dadras-Toussi et al., 2022b). As an excellent 3D printing consumable, the photosensitive resin has the advantages of high molding accuracy and short curing time and is suitable for processing precision devices (Fang et al., 2020). Although the application of photosensitive resin in the field of 3D printing has been widespread, the performance of photosensitive resin has certain defects due to the material itself. Researchers have overcome these defects through techniques such as surface modification and doping (Fleck et al., 2021; Hua et al., 2021). Mahmoudi et al. (2021) reported a photo initiator-free process for the 3D printing of a pure commercial epoxy polymer, without any resin modification (Huang et al., 2022b). A novel radical-free/cationic hybrid photosensitive resin was fabricated by a cationic curing mechanism with the process of UV-curing. It was designed and proved as a low-cost one-step printing process. With chemical modification, NR was transformed to photosensitive NR (PNR), which was blended with a commercial resin (CR) at various rubber contents (0–3 wt%) by a simple mixing approach. The synthesized photosensitive natural rubber could be used as a toughness modifier employed in ultraviolet-curable resin for the light-based 3D printing technology (Shan et al., 2020).

## 3 Bioprinting cell selection

### 3.1 Mesenchymal stem cells

Human bone marrow mesenchymal stem cells can be isolated from adult mesenchymal tissue, which has been proven of good proliferation potential. It will not lose its multi-directional differentiation ability within several generations and is widely applied as one of the ideal cell sources for cartilage tissue engineering (Tessanan et al., 2021). Recently, more and more studies have been conducted on the chondrogenic potential of MSCs in bone marrow, adipose, synovium, periosteum, umbilical cord, and muscle (Fitzpatrick et al., 2021; Tessanan et al., 2021). Plenty of promising therapies based on mesenchymal stem cells have been developed for the regeneration of cartilage defects. Synovial MSCs (SMSCs) possess strong articular specificity and chondrogenic differentiation ability. A chitosan hydrogel/3D-printed poly (ε-caprolactone) hybrid containing SMSCs and recruiting tetrahedral framework nucleic acid was developed for the cartilage regenerative system (Fazal et al., 2021; He et al., 2021). MSCs have been applied in the treatment of osteoarthritis as seed cells to rescue the defect and chronic inflammation in the joint. Bone marrow-derived mesenchymal stem cell (BMSC)-laden 3D-bioprinted multilayer scaffold with methacrylate hyaluronic acid (MeHA)/polycaprolactone incorporating ketogenic and β-TCP for osteochondral defect repair within each region. Besides, MSCs have been taken as bio-ink cells for bone regeneration. Bone tissue engineering scaffolds with MSCs can be precisely fabricated with SLA, SLM, and STL technologies (Su et al., 2021).

### 3.2 Chondrocytes

At present, chondrocytes are mainly used in the field of cartilage bioprinting (De Angelis et al., 2021). In 1994, Brittberg first introduced autologous chondrocyte transplantation (Li et al., 2021c). During the arthroscopy of the patient, healthy chondrocytes would be removed from the patient’s injured knee, while the chondrocytes were injected into the patient’s defect with 14–21 days of culture (Diloksumpan et al., 2020). Autologous chondrocyte transplantation significantly reduced swelling and pain in patients. Biodegradable waterborne polyurethane (WBPU) was modified using a water-based green chemistry process to form the ability for 3D printing. The flexibility of this material endows great compliance with tissue in the fixation of scratching wounds (Dudman et al., 2020). Silk fibroin as a natural polymer fabricated with glycidyl-methacrylate (Silk-GMA) was demonstrated for digital lighting processing 3D printing. New cartilage-like tissue and epithelium were found surrounding transplanted Silk-GMA hydrogel (Feng et al., 2020). Cell-laden alginate hydrogel containing chondrocytes was injected into 3D PCL hybrid scaffolds to support the mechanical properties of the regenerating auricle cartilage (Jang et al., 2020). The above examples illustrate that the implantation of chondrocytes can promote the repair of cartilage defect tissue.

### 3.3 Embryonic stem cells/induced pluripotent stem cells

Embryonic stem cells (ESCs) can be induced to differentiate into mesenchymal stem cells and chondrocytes and are often used in cartilage tissue engineering (Hong et al., 2020). Gene expression and immunostaining analysis confirmed that this co-culture system could form the cell colonies and secrete extracellular matrix (ECM) containing glycosaminoglycan (GAG) (Abdelrahman et al., 2022). The dynamic expression of chondrocyte-specific genes was observed during the cell monolayer expansion in this co-culture system, fully confirming the chondrogenic differentiation of human embryonic stem cells (hESCs) (Edgar et al., 2020). Fiber impedimetric responses associated with the bioinks that contained differentiated mESCs were fabricated with 3D bioprinting. Multifunctional fiber impedimetric sensors enabled the classification of stem cells with differentiation marker expression (Elsafi Mabrouk et al., 2022). Human pluripotent stem cell (hPSC)-based approach to generate organoids that interact with vascular cells in a spatially determined manner. Custom designed 3D printed microfluidic chip was applied for spatial interaction between organoid and vasculature with a matched co-culture system. Studies have shown that during the co-culture of hESCs and chondrocytes (Chds), morphogenetic factors secreted by chondrocytes can induce hESCs to differentiate into the chondrocyte lineage (Haring et al., 2020).

## 4 Application in bone and cartilage repair

### 4.1 Supporting structure

To construct personalized regenerative articular cartilage tissue, precise control of the shape and internal structure of the scaffold is crucial (Bliley et al., 2022). 3D printing technology can print a variety of bio-inks containing different biological materials, cells, and bioactive factors to construct 3D scaffolds with complex anatomical structures. Rastogi et al. reported alginate hydrogel loaded with chondrocytes and osteoblasts used 3D printing technology to construct a non-uniform hydrogel scaffold (Han et al., 2021). 3D scaffolds with different pore sizes and elastic modulus were obtained by changing the spacing and angle of the printed lines. To mimic the osteochondral structure, the formation of different tissues was observed in different locations of the same scaffold after 6 weeks of subcutaneous transplantation (Rastogi and Kandasubramanian, 2019).

In addition, the co-printing of multiple bioinks also provides a good platform for constructing the interstitial structure of articular cartilage (Piras and Smith, 2020). Articular cartilage shows differences in composition and mechanical properties from top to bottom. The construction of such heterogeneous 3D structures is difficult to achieve by traditional tissue engineering methods (Fazal et al., 2021). The Nakamura, A. team used gradient bioprinting to control cell density distribution in the same scaffold and precisely controlled the cell density by changing the mixing ratio of cell-free bioink and cell-loaded bioink at the printing needle (Jiang et al., 2021). The chondrocytes from different interstitial spaces were extracted, and these 3 cells were printed layer by layer to form interstitial structures. The experimental results showed that chondrocytes from different sources could produce specific interstitial ECM. In addition to cell selection and control, studies have shown that providing cells with appropriate biological signals or ECM components can stimulate cells to develop a zonal phenotype (Fan et al., 2020). For example, adding chondroitin sulfate and metalloproteinase-sensitive peptides to PEG hydrogels can induce MSCs to secrete external ECM components, while doping chondroitin sulfate or HA alone can induce cells to produce intermediate and deep ECM components^[^ (Nakamura et al., 2021; Beh et al., 2021). Therefore, in future research, printing different biomaterials and cells from different sources in different combinations to simulate the articular cartilage structure’s physiological function and mechanical properties will become a research direction for tissue engineering to repair articular cartilage.

### 4.2 Mechanical support

To simulate the mechanical properties of articular cartilage, many studies have used highly elastic hydrogels to construct 3D scaffolds by 3D printing to simulate the mechanical properties of joints (Mahmoudi et al., 2021). Shan et al. (2020) used a natural polymer, alginate, reinforced with an extracellular matrix derived from decellularized tissue (rECM) for 3D bioprinting. Depending on the curing time, the elastic modulus of the scaffold can be adjusted from 73.2 kPa to 40 MPa, and the scaffold has good elastic recovery, which can match the MR of the natural articular cartilage mechanical properties.

Co-printing thermoplastic materials with hydrogel materials with weak mechanical properties is also a common way to improve the mechanical properties of cartilage repair scaffolds (De Santis et al., 2021). The thermoplastic material is used as the scaffold’s skeleton to withstand the primary mechanical stress. The research shows that the mechanical properties of the hybrid printed scaffold are similar to those of pure thermoplastic scaffolds. Monfared et al. presented a dual cross-linkable hydrogel ink composed of PEG star polymer and TEMPO-oxidized nanocellulose fibers (CNFs). Shortly, hydrogels with Young’s modulus between ∼10 and 30 kPa were obtained just by altering the CNF and Ca^2+^ content. The experimental results showed that factors such as the direction and spacing of the bioink printed lines would affect the mechanical properties of the final scaffold (Park et al., 2021). Therefore, in addition to the mechanical properties of the material itself, proper printing settings and structural design also affect the mechanical properties of the scaffold (Monfared et al., 2021; Shin et al., 2021).

### 4.3 Induction of cell function

**FIGURE 7:**
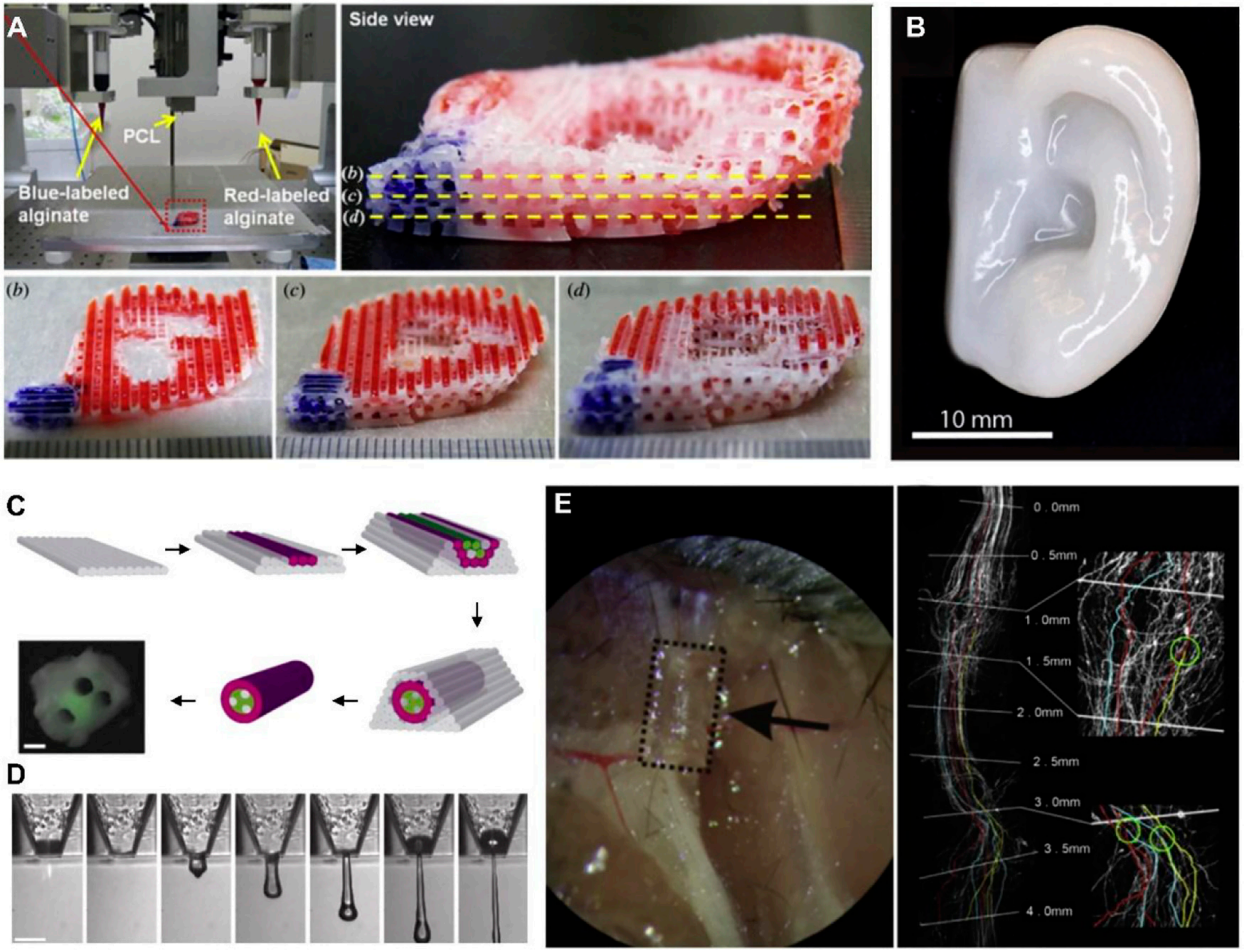
3Dioprinted tissues and organs with cell adhesion. **(A)** Printed ear-shaped PCL and alginate scaffolds **(B)** Cartilaginous ear scaffolds printed with human chondrocytes **(C)** Fabrication of a synthetic nerve graft by printing Schwann cells and BSMC **(D)** 3D printing mouse ganglion and glial cells. **(E)** Printed PEG-based guidance conduits for nerve repair. Reproduced with permission from ref (Liu et al., 2020).

Bioactive structures are constructed based on 3D printing technology. Various components with biological regulation functions, such as growth factors, proteins, peptides, drugs, and ECM components are usually doped into bioinks (Soman and Vijayavenkataraman, 2020). The ideal bone repair polychole should have a large hole with a large number of pores with particular pores greater than 100 μm. The large hole allows cells to migrate inside the bracket, promotes the integration of frames and host tissues, and guides new bone and blood vessels to grow in the shelf; the microphone can adsorb the protein on the surface of the material and affect cell proliferation, differentiation, and other behaviors through interaction with cell protein (Barreiro Carpio et al., 2021; Potyondy et al., 2021). Increased pore rate will increase the permeability and degradability of calcium phosphate-based biological ceramic porous stents, which will help cells’ attachment, proliferation, and differentiation. Still, it will reduce the mechanical strength of the bracket. Therefore the degree and mechanical strength of calcium phosphate-based biological ceramic porous brackets are still quite challenging (Ghorbani et al., 2021). The whole structure of calcium phosphate-based biological ceramic porous stents determines their mechanical strength and natural characteristics. Therefore, precisely controlling the porous bracket’s hole structure is an essential prerequisite for preparing excellent bone repair porous brackets. TGF (transforming growth factors)-family factors are often doped into hydrogel bioinks to induce chondrogenic differentiation of MSCs. Macromolecules constituting the natural cartilage matrix, such as HA, are a kind of cartilage-inducing ideal biomaterial (Murphy et al., 2019). Adding these natural macromolecules can improve the rheological properties and printing properties of bioinks (Davidson et al., 2021). It is worth noting that when using bioactive factors to induce cells to play biological functions, attention should be paid to the printing conditions for these Influence factors (Gurlin et al., 2021; Jin et al., 2021). As bio-ink and thermoplastic materials are co-printed, the high-temperature printing conditions of thermoplastic materials may affect the biological activity of factors. Post-printing modification and other methods are needed to avoid the phenomenon of factor inactivation (Figure 7) (Liu et al., 2020).

In addition to adding various biological signals, decellularized extracellular matrix (dECM) bioinks have gradually attracted the attention of researchers in recent years (Mandrycky et al., 2016). dECM is similar to natural ECM in composition and topology and can enhance cell-matrix interaction and provide a suitable microenvironment for cell growth and differentiation (Fazal et al., 2021; Neufurth et al., 2021; Suarez-Martinez et al., 2021). Obtaining dECM from various tissues to prepare bio-ink and then using 3D printing technology to build a highly open and porous 3D structure to promote the exchange of nutrients inside and outside the scaffold has become a new strategy for cartilage tissue repair (Messaoudi et al., 2020). Through 3D printing, they were printed as a single-material scaffold or a high-mechanical-strength scaffold mixed with PCL. The experimental results show that dECM derived from cartilage and fat can provide a suitable growth environment for MSCs, effectively inducing MSCs to differentiate into cartilage (Tang et al., 2020).

## 5 Conclusion and outlook

With the development of research and application of 3D bioprinting in bone and cartilage tissue engineering, researches in bioink materials have shifted from pure natural materials such as collagen and alginate to the modification of synthetic materials such as polylactic acid. Surface modification, composite with other materials would benefit the biomechanical strength, biocompatibility, immunogenicity, degradation performance, and other biological safety of biological materials. Most of the previous studies focused on the mechanical properties of materials, while recent researchers are beginning to pay attention to the principles of bionics to better construct ideal scaffolds. With the deepening of research, some problems have been revealed: how to improve the vitality of cells after printing, and how to further improve the printing accuracy based on existing technology. In recent years, with the advancement of 3D bioprinting technology, the repair of cartilage defects has been further refined. Now it is realized that articular cartilage and subchondral bone are a complete functional unit.

With 20 years of development, 3D bioprinting has achieved many gratifying achievements. It provides a fast and accurate scaffolding platform for tissue engineering to repair articular cartilage. Simulating articular cartilage with polymers and cells provides new therapeutic strategies for the repair of articular cartilage damage. However, the bioprinting of bone and cartilage is still in the preliminary stage, with many problems to be solved. Solutions for cartilage tissue build nutrient blood vessels, methods for printed cartilage tissue adapt to the biological properties of the body, and the precise distribution of cartilage cells in the scaffold according to the prefabricated tissue structure are all future research directions. It is believed that 3D bioprinting technology will have a bright future shortly (Zhang et al., 2021c).
